# Redlining, Community Wealth, and Air Pollution: A Tale of Three Cities—Boston, Nashville, and Detroit

**DOI:** 10.1007/s11524-025-01052-0

**Published:** 2026-03-11

**Authors:** Jorja Kahn, Heike Luttmann-Gibson, Jeff Blossom, Patrick H. Ryan, Brent A. Coull, Soma Datta, Tina Hartert, Antonella Zanobetti, Sima K. Ramratnam, Eneida A. Mendonça, Paloma I. Beamer, Jocelyn M. Biagini, Rima Habre, Christine C. Johnson, Christine L. M. Joseph, Gurjit K. Khurana Hershey, Katherine Rivera-Spoljaric, Akihiro Shiroshita, Edward M. Zoratti, James E. Gern, Diane R. Gold

**Affiliations:** 1 Channing Division of Network Medicine, Brigham and Women’s Hospital and Harvard Medical School, Boston, MA USA; 2 Department of Environmental Health, Harvard T. H. Chan School of Public Health, Boston, MA USA; 3 Center for Geographic Analysis, Harvard University, Cambridge, MA USA; 4 Department of Pediatrics, College of Medicine, University of Cincinnati, Cincinnati, OH USA; 5 Division of Biostatistics and Epidemiology, Cincinnati Children’s Hospital Medical Center, Cincinnati, OH USA; 6 Department of Biostatistics, Harvard T. H. Chan School of Public Health, Boston, MA USA; 7 Departments of Medicine and Pediatrics, Vanderbilt University Medical Center, Nashville, TN USA; 8 Department of Pediatrics, University of Wisconsin School of Medicine and Public Health, Madison, WI USA; 9 Division of Biomedical Informatics, Cincinnati Children’s Hospital Medical Center, Cincinnati, OH USA; 10 Asthma and Airways Disease Research Center, University of Arizona, Tucson, AZ USA; 11 Department of Community, Environment, and Policy, Mel and Enid Zuckerman College of Public Health, University of Arizona, Tucson, AZ USA; 12 Division of Asthma Research, Cincinnati Children’s Hospital Medical Center, Cincinnati, OH USA; 13 Keck School of Medicine of University of Southern California, Los Angeles, CA USA; 14 Department of Public Health Sciences, Henry Ford Health, Detroit, MI USA; 15 Department of Biostatistics & Epidemiology, College of Human Medicine, Michigan State University, East Lansing, MI USA; 16 Department of Pediatrics, Washington University School of Medicine, St. Louis, MO USA; 17 Division of Allergy, Pulmonary, and Critical Care Medicine, Vanderbilt University School of Medicine, Nashville, TN USA; 18 Department of Medicine, Henry Ford Health, Detroit, MI USA

**Keywords:** Redlining, Air pollution, Area wealth, Equity

## Abstract

Despite US air quality improvements since the 1990 Clean Air Act Amendments, disparities in air pollution between communities persist. We studied whether persistent within-city geospatial disparities in pollution continued over time in historically minoritized and under-resourced areas codified as “redlined” in 1930’s government mapping. We evaluated how longitudinal demographic patterns and disparities in community wealth and resources related to historical redlining score category in three US urban areas: Boston, MA, Nashville, TN, and Detroit, MI. We then examined longitudinal associations of redlining with changing levels of the air pollutants PM_2.5_ and NO_2_ between 2000 and 2016 in these cities. Our approach utilized daily estimates of air pollution levels from models with high spatiotemporal resolution, digitized redlining maps, and census data to evaluate temporal trends by redlining categories at the census tract level. Demographic and socioeconomic changes over time differed by city, but for each city, historically redlined areas continued to have a greater proportion of Black residents, higher poverty rates, lower income and home values, and a higher social vulnerability index (SVI). Over all areas, air pollution levels declined markedly over time, but for annual averaged NO_2_, redlining-area-associated exposure disparities persisted in Boston and widened in Nashville. In contrast, by 2016, regardless of redlining history, areas in Detroit had similar NO_2_ pollution levels. Our results highlight the lasting social, economic, and environmental effects of urban discriminatory practices, also showing that in some cities, areas may be equally exposed to specific criteria pollutants, regardless of area wealth.

**Supplementary Information:**

The online version contains supplementary material available at 10.1007/s11524-025-01052-0.

## Introduction

Despite overall improvements in U.S. air quality since the 1990 Clean Air Act Amendments, geospatially varying racial, ethnic, and socioeconomic disparities in air pollution exposures have persisted over time [1, 2] putting marginalized and minoritized communities living in more polluted areas at risk for poorer health [3, 4]. In the 1930 s following the Great Depression, the Home Owners’ Loan Corporation (HOLC) was a New Deal federal government initiative that mapped urban neighborhoods, categorizing areas into four color-coded grades in part based on the proportion of Black or immigrant persons in the neighborhood: A (“best”—green), B (“still desirable”—blue), C (“definitely declining”—yellow), and D (“hazardous”—red, i.e., “redlined”) [4, 5]. Additional mapping criteria included “the age and condition of housing, transportation access, closeness to amenities such as parks or disamenities like polluting industries, and the economic class and employment status of residents.”[6] HOLC redline mapping often reflected and codified already intrenched, but less codified preexisting patterns of targeted community marginalization, discriminatory private or governmental housing practices, and area-specific under-resourcing. Residents in redlined neighborhoods, suffering over time from private and government discrimination before, during, and after the HOLC mapping period, were often ineligible to receive mortgage assistance, limiting neighborhood investment and wealth-building ability [7–9]. Despite the ban on discriminatory housing policies with the Fair Housing Act of 1968, the legacy of discriminatory practices in minoritized areas, including those mapped as “redlined” continues, as demonstrated by lasting area/neighborhood patterns of residential segregation, low home ownership, relative absence of adequate housing, and fewer social, economic, and educational resources [6, 10–12].

Discriminatory land use decisions, along with unequal enforcement of environmental regulations and reduced economic and political power of minoritized residents, have over time influenced the spatial distribution of air pollution sources, with historically redlined areas more likely to contain pollution emission sources including industrial facilities, railroads, and major roadways [13–15]. As a result, cross-sectional studies have consistently documented higher levels of air pollutants in redlined areas [16, 17]. A recent longitudinal study demonstrated how historical redlining around New York City (NYC) schools related to the persistence of air pollution disparities between 2009 and 2018 [14].

We focused on longitudinal redlining and pollution patterns in three US cities: Boston, MA, Nashville, TN, and Detroit, MI. By city, we compared air pollution levels and trends in levels from 2000 through 2016, between areas defined by the percentage of the area mapped and codified by HOLC in the 1930 s as A, B, C, or D. Estimating exposures at the census tract level, we focused on the criteria pollutants PM_2.5_ and NO_2_. In addition to asking whether marginalized areas that historically had fewer resources and wealth persisted with higher pollution, we also assessed whether an influx of wealth and resources into these areas led to lower pollution levels.

## Methods

### Study Design

**Fig. 1:**
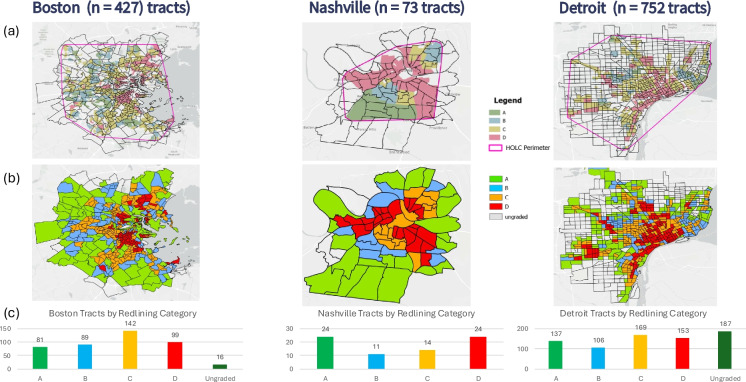
Maps of Boston, Nashville, and Detroit presenting (**a**) boundary of analysis identification, (**b**) redlining score assignment, and (**c**) distribution of tracts by redlining score category

An ecologic study design was conducted at the census tract level in three cities: Boston, Nashville, and Detroit. These three cities were selected for case histories of redlined communities that differed in their historical context and geography and were locations of birth cohorts participating in the Children’s Respiratory and Environmental Workgroup (CREW), funded by the Environmental Influences on Child Health Outcomes (ECHO) program [18, 19]. Shapefiles from the 2010 census, downloaded from the National Historic Geographic Information System (GIS) in June 2020, were used to determine the boundary of analysis for each city [20]. The smallest convex polygon that enclosed the original HOLC map (described below) was identified using the “minimum bounding geometry convex hull” tool in version 2.9 of ArcGIS Pro (Esri, Redlands, CA). For each city, the analysis includes all census tracts found within or intersecting the convex polygons (Fig. 1a).

### Redlining Assessment

Redlining maps were retrieved from publicly available digitized HOLC maps from the Mapping Inequality Project by the University of Richmond. Due to spatial misalignment between historical redlining maps and census boundaries, redlining “scores” were calculated for each census tract to evaluate the extent of historical redlining within 2010 tract boundaries. First, the georeferenced HOLC maps were downloaded in shapefile format and overlayed with the 2010 census tracts using ArcGIS. The identity and calculate geometry tools in ArcGIS Pro were used to spatially calculate the land area within each census tract that was graded and color-coded by the original HOLC map (A: “best,” green; B: “still desirable,” blue; C: “definitely declining,” yellow; D: “hazardous,” red).

The graded areas were then compared to the total land area to determine the percentage of land classified by each HOLC grade. Based on the grade percentages, a weighted redlining score was calculated for each census tract using a similar method previously described by Lynch and colleagues [21]. In brief, the percentages of tract area graded A, B, C, or D were multiplied by the numerical grade assigned to each category (1, 2, 3, or 4, respectively) and summed to a continuous score. The score was subsequently categorized into groups that corresponded to the original HOLC grading: > 0–1 (A), > 1–2 (B), > 2–3 (C), > 3–4 (D). If no area within a tract was covered by the HOLC maps, it earned a score of 0 and was assigned “ungraded” as an additional category.

### City-Specific Census and SVI Characteristics and Their Changes over Time, by Redlining

We assessed city-specific neighborhood characteristics across redlining tract-level score categories over approximately the same period (2000 through 2016) of our assessment of city-specific longitudinal changes in pollution, by redlining score category. The U.S. Census data from the start of this period were ascertained during the single year 2000. Subsequent available national census data were not that finely resolved temporally, but rather represented summaries of demographics over windows of sampling that included 2016. The demographic data from the American Community Survey (ACS) that we present is based on the 5-year sampling window of 2015–2019. Descriptive analysis included the census tract variables: total population, population density (per square mile); population percentage of White, Black or African American (census categories for race), and (from a separate distinct category for ethnicity) Hispanic or Latino residents; population percentage over 25 years old with a bachelor’s degree; percentage below the poverty level; and median household income, gross rent, and house value. Racial and ethnicity variables were self-identified, and monetary variables were inflation adjusted to 2012.

We averaged the tract values for each census demographic, social, or economic variable by redlining score by census year and then calculated the percent change over time in the score-specific demographic values between 2000 and the ACS sampling window of 2015–2019 (Table 1). Missing values of census variables were omitted from analysis; the number of observations used to calculate each variable is detailed in Fig. 1.

**Table 1:** Census/American Community Survey variables, averaged by redlining score category, city, and census year

	2000 (census year)					2015–2019 ACS window (mid-point = 2017)^b^					% change				
	Ungraded	A	B	C	D	Ungraded	A	B	C	D	Ungraded	A	B	C	D
**Boston**															
Total population	4848	4458	4290	4636	4176	4776	4540	4448	4523	4240	−1.5%	1.8%	3.7%	−2.4%	1.5%
Population density (per sq. mile)	4608	4674	9113	15,893	27,436	4641	6613	11,012	20,042	30,622	0.7%	41.5%	20.8%	26.1%	11.6%
% White	86.6	85.0	81.5	70.1	55.3	79.8	74.5	71.5	63.4	54.6	−7.9%	−12.4%	−12.2%	−9.6%	−1.3%
% Black	3.7	3.9	5.6	15.4	22.1	4.6	6.7	8.0	16.3	21.6	26.3%	70.4%	42.5%	5.9%	−2.1%
% Hispanic	3.9	4.8	6.1	8.2	17.0	8.0	9.1	12.0	13.9	22.0	103.2%	90.1%	97.5%	69.3%	29.0%
Median household income (US dollars)^a^	82,775	94,054	85,507	69,640	52,459	87,525	104,553	94,626	78,482	62,484	5.7%	11.2%	10.7%	12.7%	19.1%
% below poverty level	6.1	5.2	6.6	8.6	16.1	10.0	11.3	9.5	11.9	17.1	63.3%	117.9%	44.0%	37.8%	6.2%
Owner-occupied housing units: Median value (US dollars) ^**a**^	334,085	419,508	431,935	342,542	296,578	447,206	599,081	605,976	516,863	490,727	33.9%	42.8%	40.3%	50.9%	65.5%
Median gross rent (U.S. dollars)*	1296	1338	1300	1239	1050	1608	1661	1536	1550	1340	24.0%	24.1%	18.1%	25.1%	27.6%
% population ≥ 25 years with bachelor’s degree	22.6	24.6	23.0	22.2	17.4	29.6	29.2	26.8	26.9	25.2	31.0%	18.9%	16.5%	21.0%	44.5%
**Nashville**															
Total population		4314	4392	3755	2895		3890	4444	4360	2802		−9.8%	1.2%	16.1%	−3.2%
Population density (per sq. mile)		1862	3029	5394	4165		1926	3134	5244	4129		3.4%	3.5%	−2.8%	−0.9%
% White		72.1	67.6	59.5	34.8		73.2	62.9	71.2	49.2		1.5%	−6.9%	19.7%	41.3%
% Black		20.5	25.3	33.5	59.0		19.6	29.5	19.9	44.5		−4.3%	16.7%	−40.6%	−24.6%
% Hispanic		5.6	3.8	3.1	3.8		8.0	9.5	5.4	7.5		42.3%	151.9%	73.0%	96.6%
Median household income ^**a**^		79,070	59,241	39,150	31,393		82,910	61,553	59,918	41,165		4.9%	3.9%	53.0%	31.1%
% below poverty level		6.9	9.3	16.1	26.9		9.9	17.5	16.5	22.9		44.6%	88.3%	2.5%	−15.0%
Owner-occupied housing units: median value ^**a**^		237,257	211,883	162,944	95,994		364,645	326,185	381,160	228,367		53.7%	53.9%	133.9%	137.9%
Median gross rent ^**a**^		953	899	775	598		1198	1066	1128	848		25.7%	18.6%	45.4%	41.8%
% population ≥ 25 years with bachelor’s degree		23.0	24.0	20.4	8.5		28.5	28.0	33.3	22.8		23.8%	16.5%	63.4%	169.7%
**Detroit**															
Total population	3397	3561	3636	3305	2886	3264	3312	3080	2603	2233	−3.9%	−7.0%	−15.3%	−21.2%	−22.6%
Population density (per sq. mile)	4083	4876	6912	7215	7278	3900	4377	5601	5395	5573	−4.5%	−10.2%	−19.0%	−25.2%	−23.4%
% White	81.4	67.4	42.0	49.0	30.8	68.9	55.7	37.3	46.3	31.3	−15.3%	−17.4%	−11.2%	−5.6%	1.7%
% Black	13.4	27.3	53.5	45.8	61.1	22.3	37.4	57.2	48.0	59.6	66.8%	36.9%	6.9%	4.7%	−2.6%
% Hispanic	1.9	2.3	2.4	2.9	6.3	3.3	3.7	3.7	5.1	9.1	71.1%	62.9%	52.5%	75.8%	43.8%
Median household income ^**a**^	73,088	67,991	63,303	51,629	38,652	56,020	51,767	48,351	38,950	29,122	−23.4%	−23.9%	−23.6%	−24.6%	−24.7%
% below poverty level	5.1	7.6	14.2	15.8	22.8	11.9	16.5	22.5	25.1	33.7	134.4%	116.4%	58.8%	58.4%	47.9%
Owner-occupied housing units: median value ^**a**^	189,962	178,241	163,657	118,698	89,343	132,970	119,428	111,444	83,241	74,427	−30.0%	−33.0%	−31.9%	−29.9%	−16.7%
Median gross rent ^**a**^	1005	936	873	772	670	960	919	888	801	717	−4.5%	−1.8%	1.7%	3.7%	6.9%
% population ≥ 25 years with bachelor’s degree	14.3	13.6	12.4	9.5	6.8	16.8	16.6	14.3	11.6	10.3	17.8%	21.9%	15.4%	22.8%	51.9%

^**a**^ Inflation adjusted to 2012

^**b**^2017 is the mid-point in time for the American Community Survey (ACS) (5-year estimates; 2015–2019). For details, see Methods section

In addition, we used Social Vulnerability Index (SVI) to compare a summary of neighborhood vulnerability by redlining score over the period of interest. As with the U.S. Census data, SVI data in 2000 represented neighborhood vulnerability ascertained for that single year, whereas the SVI data that included the year 2016 (published in 2018) represented a 5-year window covering/summarizing data from 2012 through 2016. The SVI data, only released bi-annually, use census/ACS data to rank census tracts on 15 sociodemographic factors grouped into four domains to assess natural disaster preparedness: socioeconomic status (SES), household composition and disability, “minority” status and language, and housing type and access to transportation [22]. SVI values range between 0 and 1 based on their nationally normed percentile ranking, with higher values reflecting more vulnerability. We calculated the average SVI by redlining category and city for 2000 and 2012 through 2016 (Table 2), and generated box plots to visually describe the SVI distributions and compare changes in tract-level SES/SVI over time (Supplementary Fig. 1).

**Table 2:** Average Social Vulnerability Index(SVI) by redlining score category, city, and census year

	SVI Score (SD)									
	2000					2012–2016 window of observation				
	A	B	C	D	Ungraded	A	B	C	D	Ungraded
Boston	0.332 (0.234)	0.357 (0.254)	0.481 (0.273)	0.664 (0.249)	0.328 (0.232)	0.348 (0.219)	0.355 (0.268)	0.445 (0.268)	0.598 (0.286)	0.314 (0.174)
Nashville	0.400 (0.348)	0.481 (0.351)	0.613 (0.251)	0.809 (0.202)		0.343 (0.348)	0.449 (0.310)	0.330 (0.309)	0.741 (0.211)	
Detroit	0.441 (0.269)	0.558 (0.326)	0.606 (0.317)	0.777 (0.229)	0.334 (0.217)	0.523 (0.283)	0.601 (0.320)	0.636 (0.305)	0.779 (0.220)	0.449 (0.252)

### Area-Specific Air Pollution Estimation by Redlining

For the three cities, ambient daily PM_2.5_ and NO_2_ pollution levels 2000–2016 were estimated at the census tract level using 1-km^2^ data from previously described and validated spatiotemporal models [23, 24]. Briefly, the models integrate multiple machine-learning algorithms to generate daily estimates of ambient pollution concentrations on a 1-km^2^ grid across the contiguous United States by incorporating multiple predictor variables, including satellite measurements, ground-based monitoring, and meteorological variables.

To evaluate air pollution exposures at the census tract level, each tract was assigned the area-proportional weighted air pollution estimate from all grid polygons that overlay any part of the tract. To perform this calculation, we used the ArcGIS Pro “intersect” tool between the tract polygons and air pollution grid polygons. The area was then re-calculated for these overlapping areas and divided by the total tract area. This produced the percentage (weight) of the tract within each air pollution grid area. These weights were applied to the air pollution values and summarized at the tract level to produce the tract level exposures.

**Fig. 2:**
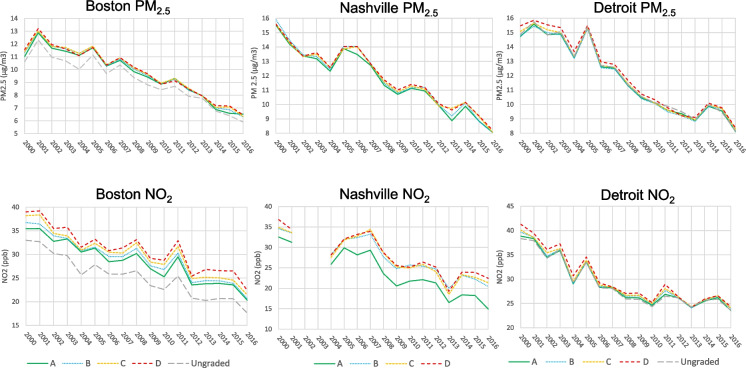
Longitudinal trends of average annual PM_2.5_ and NO_2_ by redlining score category between 2000 and 2016

Data cleaning procedures were performed to identify pollution data outliers and verify the correct linkage of all tracts to their corresponding air pollution grid points. Missing values of variables, occurring sporadically, were omitted from analysis. For data visualization, annual air pollution estimates were averaged within each redlining score category (A, B, C, D, ungraded) for each city between 2000 and 2016. Based on the daily estimates, annual averages of PM_2.5_ and NO_2_ were calculated for each census tract. For NO_2_ monitoring data for Nashville, we observed clusters of aberrant low values throughout 2002 and 2003 (Supplementary Methods) with no concurrent drop in PM_2.5_ measures or historical evidence of a reason for the outliers (Supplementary Table 1a and 1b). As they unduly influenced our grade-specific and overall annual means for those 2 years, we removed them from our analyses (Fig. 2).

### Statistical Analysis

Separately for each city, we applied linear mixed-effects models to evaluate associations between redlining categories and annual PM_2.5_ and NO_2_ levels and trends over time. The models contained redlining score category (reference = A category) and calendar year (a linear time trend) as main effects, and an interaction term score category*year to allow the time trends (slopes) to vary across score categories. This enabled us to evaluate (1) the overall effect of redlining score on the annual pollution levels and on the slopes based on the 16 years of observation; (2) the pairwise difference of annual levels or slopes in *each* of the redlining scores D, C, B or ungraded, compared to A. The models also contained random effects to account for longitudinal correlation that arises from the fact that repeated pollution measures were recorded for each census tract. As our primary results, we report the estimated annual PM_2.5_ and NO_2_ levels and the estimated (from the mixed model) slopes over the period 2000–2016 by redlining score category. A Bonferroni correction was applied to control for multiple testing across pairwise redlining score category comparisons (each of D, C, B versus A), separately for pollution levels (the main effects of score category) and time trends (the time × score category interaction) (Table 3). For completeness, we also present the mean annual pollution levels for each score category and year, as well as the standard errors of these means, in supplemental material (Supplemental Tables 1a and 1b).

**Table 3:** Estimated^a^ PM_2.5_ and NO_2_ exposure levels by redlining score category, city, and beginning, middle, and ending years during 2000–2016

	Redlining score		Estimated average annual PM_2.5_ (μg/m^3^)			
City	category	***N***_tracts_	2000	2008	2016	Slope
			(*p* = 0.016)^b^	(*p* = 0.002)^b^	(*p* = 0.014)^b^	(*p* = 0.2912)^c^
Boston	D	99	12.8	9.8	6.9	−0.37
	C	142	12.8	9.9	6.9	−0.37
	B	89	12.8	9.8	6.8	−0.38
	Ungraded	16	12.0	9.2*	6.5	−0.34
	A (ref)	81	12.6	9.7	6.8	−0.36
			(*p* = 0.819)^b^	(*p* = 0.015)^b^	(*p* = 0.001)^b^	(*p* = 0.2017)^c^
Nashville	D	24	15.1	11.9*	8.8*	−0.39
	C	14	15.1	11.9	8.7*	−0.40
	B	11	15.1	11.9	8.6	−0.41
	A (ref)	24	15.0	11.7	8.5	−0.41
			(*p* < 0.0001)^b^	(*p* < 0.0001)^b^	(*p* = 0.410)^b^	(*p* < 0.0001)^c^
Detroit	D	153	16.2*	12.1*	8.1	−0.50*
	C	169	15.8	12.0	8.1	−0.49
	B	106	15.6	11.8	8.0	−0.47
	Ungraded	187	15.8	11.9	8.1	−0.48
	A (ref)	137	15.7	11.8	8.0	−0.48
			Estimated average annual NO_2_ (ppb)			
			(*p* < 0.0001)^b^	(*p* < 0.0001)^b^	(*p* < 0.0001)^b^	(*p* = 0.039)^c^
Boston	D	99	38.0*	31.1*	24.2*	−0.86
	C	142	37.2*	30.0*	22.8*	−0.90
	B	89	35.9	29.0	22.1	−0.86
	Ungraded	16	31.7*	25.2*	18.6*	−0.82
	A (ref)	81	35.0	28.3	21.5	−0.84
			(*p* = 0.001)^b^	(*p* < 0.0001)^b^	(*p* < 0.0001)^b^	(*p* = 0.042)^c^
Nashville	D	24	35.7*	28.5*	21.3*	−0.90*
	C	14	35.3*	28.0*	20.6*	−0.91
	B	11	35.1	27.7*	20.2*	−0.93
	A (ref)	24	33.5	24.5	17.4	−1.01
			(*p* < 0.0001)^b^	(*p* < 0.0001)^b^	(*p* = 0.001)^b^	(*p* < 0.0001)^c^
Detroit	D	153	37.9*	30.2*	22.4	−0.96*
	C	169	36.9*	29.7*	22.4	−0.91
	B	106	36.5	29.4	22.3	−0.88
	Ungraded	187	36.0	29.0	22.1	−0.87
	A (ref)	137	36.3	29.2	22.1	−0.88

^a^ Predicted values from linear mixed model containing main effects of redlining score category, calendar year, and (score category*year) interaction

^b^ *p*-value from overall ANOVA test of year-specific differences in PM_2.5_ or NO_2_ concentrations across redlining score categories (from linear mixed model)

^c^ *p*-value for overall ANOVA test of differences in linear slopes over time, across redlining score categories (from linear mixed model)

^*^ Denotes significance of a pairwise comparison between areas in this group and those in the A group, based on a Bonferroni adjustment (*p* < 0.05/4 (BOS, DET), *p* < 0.05/3 (NASH)), from linear mixed model

## Results

### Redlining Score Assignment

The analysis included a total of 1252 census tracts: 427 tracts in Boston, 73 tracts in Nashville, and 752 tracts in Detroit (Fig. 1a). As a tract is defined by population and not geography, our three cities varied in terms of the geographical size of the tracts, with average tract sizes (2010) for Boston, Detroit, and Nashville of 0.68, 0.77, and 1.63 mi^2^, respectively. The classification of census tracts based on the redlining score method exhibited spatial patterns similar to those observed in the original HOLC maps (Fig. 1b). Across the three cities, redlined D areas were typically concentrated in the urban center. Although the score distribution varied by city, we identified an even distribution of tracts across the A-D categories, with no single score category representing more than a third of tracts (Fig. 1c). Due to the shapes of their original HOLC maps, the boundary analysis for Boston included fewer ungraded tracts (*n* = 16, 3.7%) and none in Nashville. That is, the convex hull of the graded areas in Nashville did not contain any ungraded area. In contrast, the tracts scored as ungraded were the largest group in metropolitan Detroit, with 187 tracts (24.9%).

### Neighborhood Characteristics

Overall, demographic and socioeconomic gradients reflected the redlining score (A, B, C, or D) for all three cities (Table 1). In all three cities, during both census periods shown in Table 1, formerly redlined (D) neighborhoods had the lowest proportion of White residents, highest proportion of Black residents, and lowest percentage of college-educated residents. Similar patterns were observed for the variables of median household income, home value, and gross rent, all of which were the highest in A-scored tracts and lowest in D-scored tracts. In both census time periods, across all three cities, redlined D tracts displayed higher social vulnerability (SVI) compared to A-scored and ungraded tracts, with mean SVI scores approximately twice as high (Table 2, Supplementary Fig. 1).

However, we did observe city-specific shifts in demography and wealth over time. In 2000, median household income was 1.8 times higher for Boston A ($94,054) compared to D ($52,459) areas, and 2.5 times higher for Nashville A ($79,070) compared to D ($31,393) areas. While substantial income disparities persisted between A and D areas in 2000 and the later time window (2015–2019), over this period, both Boston and Nashville experienced within A and within D areas growth in household income and home values. In contrast, over time within each of the graded Detroit areas (A, B, C, D), we observed declining incomes and home values, as well as increasing rates of the percent of the population below the poverty level, rising in Detroit from 7.6% to 16.5% for A areas, and from 22.8% to 33.7% for D areas (Table 1).

In the Boston D tracts, the proportion of Black and White residents changed little between 2000 and 2017 (22% and 55%, respectively, for both periods). Compared to Boston or Detroit D areas, Nashville’s D areas had the largest percent increase in median income (+ 31%), accompanied by a drop in the proportion of Black residents (− 24.6%).

### Air Pollution Levels and Trends by City and Redlining

Figure 2 presents the annual averages across all years (2000 through 2016), by redlining score category, with numerical values plotted in this figure and presented in Supplemental Table 1a. and 1b. While the estimated slopes presented in Table 3 were estimated from linear mixed models (LMM) using all years of pollution data 2000–2016 (Supplementary Tables 1a and 1b), in this table, we present the estimated, redlining score category-specific mean air pollution levels from this model (hereafter, LMM-estimates) for sample years that represent the beginning, the middle, and the final year of our study: 2000, 2008, and 2016. Table 3 also shows (1) the significance of the LMM-estimated differences of air pollution *levels* for each score category (D, C, B, ungraded) compared to A tracts for each of these three time points, city, and pollutant; (2) the LMM-estimates of the redlining-score specific slopes of the changes in air pollution over time by city and pollutant; and (3) the significance of these LMM-estimated differences in slopes by city and pollutant.

For comparison of pollution levels or slopes within the city, between redlining score categories, we first evaluated the overall ANOVA-type test. Then, only in the event that the overall test was significant, adjusting for multiple comparisons using a Bonferroni adjustment, we then made pairwise comparisons to evaluate whether each of the redlining scored area levels (B vs. A, C vs. A, D vs. A) differed from the A group. We show both the significance of the overall tests and the pairwise comparisons in Table 3.

For all three cities at the onset of observation (2000), the annual mean NO_2_ levels were significantly higher for the D and C redlining-scored areas, when the pollutant levels in each of the B, C, and D areas were compared to that in the A areas (Fig. 2; Table 3). Over all areas, regardless of redlining score category, PM_2.5_ and NO_2_ levels declined over the period 2000–2016. In all three cities, between 2000 and 2008, compared to A areas, D and C areas remained more polluted with NO_2_. However, by 2016, while D areas continued to have higher annual averaged NO_2_ levels than A areas in Boston and Nashville, 2016 NO_2_ levels were similar across areas in Detroit (Fig. 2; Table 3).

### Slope of Decline in Air Pollution (2000 to 2016) by City and Redlining Grade

In Boston, the slopes of decline in NO_2_ for each of the redlining scored areas were similar, ranging from − 0.90 to − 0.82 (Fig. 2; Table 3). In Nashville, the annual decline in NO_2_ was greater for the A areas than for the D areas, with D slopes diverging from A slopes (pollution getting less improved for D compared to A areas) (Fig. 2, Table 3). In Detroit, NO_2_ levels declined more in D than A areas, and by 2016, levels of the two pollutants were similar across all redlining categories (Table 3).

Although all three cities experienced an overall decline in both PM_2.5_ and NO_2_ over the 17-year period, pollution levels and rates of decline varied by redlining score category and city (Fig. 1). Averaged across all score categories for the year 2000, PM_2.5_ concentrations were 11.3 (SD = 1.1) μg/m^3^ in Boston, 15.7 (0.4) μg/m^3^ in Nashville, and 15.0 (0.8) μg/m^3^ in Detroit, while NO_2_ concentrations were 37.3 ppb (3.8), 34.9 (2.2) ppb, and 39.6(2.4) ppb, respectively. By 2016, PM_2.5_ levels had declined to 6.4 (0.7) μg/m^3^ in Boston, 8.2 (0.4) μg/m^3^ in Nashville, and 8.3 (0.9) μg/m^3^ in Detroit, while NO_2_ concentrations declined to 21.3 (3.1) ppb, 20.3 (4.2) ppb, and 23.9 (1.2) ppb, respectively.

## Discussion

As others have demonstrated in cities throughout the USA over the period following the EPA Clean Air Acts [25], we saw marked overall city-wide reductions in PM_2.5_ and NO_2_ annually averaged levels from 2000 through 2016 in Boston, Nashville, and Detroit. However, within these cities, between-area pollution disparities persisted. Urban areas in the lowest redlining score category (A) were exposed to higher NO_2_ levels compared to areas with historical redlining (D and C) (Table 3). The most consistently significant between-area differences in NO_2_ levels occurred in the first half of 2000–2016. Between-area within-city differences in PM_2.5_ were less consistent. Longitudinal patterns in the magnitude and duration of NO_2_ pollution disparities were city-specific. Over the 2000–2016 period in Boston, disparities in NO_2_ levels in D-scored compared to A-scored areas remained relatively constant over time. In contrast, in Detroit the gap in NO_2_ levels between historically D-scored and A-scored areas decreased, and in Nashville, the gap in NO_2_ levels increased.

### Deindustrialization with Urban Renewal: Detroit

In addition to the persistence of redlining-based disparities in resources and economic opportunities including home ownership, the census data confirm that in all areas, central Detroit experienced an overall population decline with a corresponding loss of income, increasing rates of poverty, and decreasing home values [26]. This likely reflects the progressive decentralization, relocation, and decline of the local auto and associated industries to suburban areas and the loss of local resources and industry-related jobs that had begun prior to the 2000–2016 period that we studied [27]. New economic resources to revitalize central Detroit resulted in two new sports stadiums, new restaurants and service industries, and some new housing but not necessarily in new industry or employment opportunities adequate to support local communities like the auto industry had done [28]. Against the background of regional (from the USA or Canada) pollution sources, a reduction in redlined-area–specific industrial and other local sources, the hollowing out of many neighborhoods, combined with new traffic patterns may have resulted in a more even city-wide dispersion of traffic-related pollution [26].

### Do Redlined Area-Level Increases in Wealth Lead to Pollution Reduction? Boston vs. Nashville

In contrast to Detroit, Boston and Nashville experienced an increase in the proportion of college-educated individuals, median household income, and home values in historically redlined areas. Boston Metropolitan neighborhood demographic, housing, and income changes during the decades of interest in our report were mapped interactively and described in detail in a Harvard University Joint Center for Housing Studies report. The report used the U.S. Census data to summarize the complex Boston demographic changes between 1990 and 2016 as including “(1) growing racial and ethnic diversity but continued isolation within the region; (2) increased affluence but rising levels of income inequality; (3) somewhat more concentrated poverty; (4) declining amounts of modest-cost housing; (4) rising numbers of cost-burdened renters and homeowners; and (5) gentrification or stagnation in low-income tracts” [29]. The continued isolation that they describe in Boston resulted in part from post-1930s “block-busting” and other discriminatory practices that reinforced continued area-specific segregation of Black and other minoritized communities in historically redlined areas [12].

Overlapping with demographic change, between 1991 and 2006/7, the “new” Central Artery/Tunnel “Big Dig” that depressed high burden traffic in parts of Boston was completed. Its effects on neighborhoods and beneficial changes in near-roadway pollution were superimposed on effects of many remaining very old established transportation patterns [29]. The greatest density of traffic and pollution sources remained in central Boston, with its major highways, bus routes, local road traffic, railroad stations and lines (both commuter and freight), and nearby city port and airport activities. Thayer and colleagues have shown evidence of inequitable distribution of near roadway ultra-fine particle concentrations in central Boston [30]. However, they also point out that over time pockets of central Boston have developed with a “concentration of high-income housing coincident with high pollution levels.” In the midst of these demographic and transportation pattern changes in Boston, our data show that the relative gap in pollution between historically redlined and non-redlined neighborhoods remained similar from 2000 through 2016. This pollution disparity may have contributed to persistent health inequities [31–33].

In Nashville, we observed a widening difference in pollution between historically redlined and non-redlined areas over time. While Nashville is historically a railroad hub, and was located near coal-fired power plants, traffic appears to be the growing source of air pollution, with some highways traveling through urban neighborhoods [34]. The well-documented inflow of new wealth, entertainment and service industries to Nashville may have brought new traffic and other NO_2_ sources to historically redlined areas [35]. Simultaneously, the proportion of Black residents in those areas decreased, possibly due to displacement as housing grew less affordable (as documented by the Nashville Affordable Housing Task Force), or as work for long-time residents became less available [36, 37].

### Area and City Differences in Which Criteria Pollutants Are Linked to Historical Redlining

The specific mix of air pollutant exposures linked to historical redlining varies by the specific mixtures of local pollution sources, patterns of roadways, siting of local industrial sources, and city-wide and regional contributions to exposures, as well as the method of measurement or estimation of pollutant levels [10, 38]. Thayer, Brugge and colleagues have shown the added insight gained by measuring near-roadway pollution that may not be among the criteria pollutants used by the EPA for regulation [30]. Studying the vicinities around NYC schools from 2000 to 2018, Jung and colleagues found that while pollution trended lower overall, the reduction over time in pollution was smaller for schools in redlined areas [14]. However, in NYC, the effects of redlining were seen specifically for PM_2.5_, traffic-related PM components (black carbon), and the primary emission NO but not for NO_2_ [14].

### Health Significance of City-Wide and Between-Area, Within-City NO_2_ and PM_2.5_ Levels

Being exposed to NO_2_ and PM_2.5_ at the overall and area-specific levels that we estimated for Boston, Nashville, and Detroit has clinical relevance. In Project Viva, a Boston birth cohort studied during the same period (2000–2016), lower levels of lung function and greater airway inflammation (FENO) in teens were associated with higher NO_2_ exposures averaged over the first year of life and up to the teen years [39]. The median NO_2_ exposures in that study were 33.1 ppb (interquartile range [IQR], 10.4) and 24.5 ppb (IQR, 8.9 ppb) through early teens. In a 2000–2016 study of Medicare beneficiaries throughout the contiguous USA, higher levels of NO_2_ and PM_2.5_ were associated with more hospitalizations for cardiovascular disease, coronary heart disease, and cerebrovascular disease [40]. Associations were stronger at the lower end of the exposure distributions, which for NO_2_ was a median level of 10.6 ppb (IQR, 5.6 ppb). No threshold has been found for these adverse pollution health effects. These and other studies suggest that by 2016, while NO_2_ levels had improved, they still were relevant for health effects. For example, an annual average of 21.6 ppb in Nashville for the D areas has potential clinical relevance, as does the significant difference in annual average ppb exposures between the D and the A areas.

### Study Strengths and Limitations

Our study contributes a longitudinal perspective to the literature, demonstrating cross-sectional city-specific disparities in air pollution exposures for historically redlined neighborhoods [10, 14, 16, 17]. While our findings may not be fully generalizable to other redlined U.S. cities, some lessons learned may be relevant. Our study is limited by a focus on two regulated criteria pollutants, PM_2.5_ and NO_2_, without complementary area-level data on toxic particle-level components or other gases that may have local sources. Cross-sectional city-specific studies have demonstrated that historically redlined communities have also had disparities in the traffic particle component black carbon, ultrafine particles, volatile organic compounds, and sulfur dioxide [11, 16, 17]. Nevertheless, the redlining-based pollution disparities we describe have high relevance to health risk [11, 16, 41, 42]. Higher air pollution exposures to either PM_2.5_ or NO_2_ at levels below EPA regulatory thresholds can lead to greater risk of adverse respiratory and cardiovascular outcomes [43–45].

While we did not have detailed longitudinal data on pollution sources to account for the city-specific trends and changes in pollution within and between areas with A through D redlining grades, the models estimating PM_2.5_ and NO_2_ over time took into account the change in those sources. The overall decline in air pollution in the past several decades (recently slowed in some parts of the country by the increase in pollution from wildfires [46]) is generally attributed to environmental (including traffic) regulation, enforcement of pollution controls, and deindustrialization. Superimposed are regional and local influences on pollution, some constant (geography) and others changing over time—climate, urban land use, siting of polluting industries, roads, traffic policies, public and private vehicle emissions controls. These factors merit study for defining opportunities to reduce urban pollution.

One challenge in our geospatial approach was alignment between historical HOLC maps, modern census tracts, and 1-km^2^-grid air pollution estimates. To conduct our analysis at the census tract level, we calculated a redlining score, which may have contributed to potential misclassification of redlining exposure. For Boston and Detroit, we also created an “ungraded” category representing locations within our urban perimeter not graded by HOLC maps that, at the time of HOLC mapping, represented non-residential areas (e.g., rural or sparsely populated areas or industrial or commercial areas). In our Boston and Detroit data, compared to historically redlined D areas, ungraded and A-graded areas had higher wealth and lower NO_2_ air pollution. As they were estimated from well-validated spatio-temporal prediction models, we acknowledge there is some error associated with the predicted pollution levels analyzed in this study. However, the magnitude of this error is certainly much less than that reported in those modeling efforts [47], as we analyze levels averaged over both space (census-tract) and time (annual averages).

We relied on census-based classifications of race, ethnicity, and socioeconomic indicators, but other area-level measures reflect structural inequities, some but not all of which may be linked to historical redlining. The census may undercount some marginalized populations. Persistent racial segregation and under-resourcing of minoritized communities with siting of polluting industries in redlined D neighborhoods have also resulted from other policies and practices, some existing long before HOLC map development. As a well-documented federally sponsored housing policy enacted across the country with accessible records and explicitly racist determinations, redlining associations with air pollution are easier to document than the effects of racial covenants or other discriminatory policies more difficult to measure or geographically localize. In a nationwide study, Lane and colleagues found that while air pollution disparities were larger by redlining category than by race and ethnicity, racial and ethnic disparities in pollution exposures persisted within each HOLC category [10]. Defining these disparities may require more spatial resolution—at the block rather than the tract level.

## Conclusion

Against a background of overall reduction in annual PM_2.5_ and NO_2_ pollution, in Boston, Nashville, and Detroit, we identified measurable city-specific differences in longitudinal trends in disparities in pollution levels between historically redlined and non-redlined areas. Deliberate city planning efforts will be needed to assure reduction of pollution exposures and increases in local healthy housing and job opportunities across all city areas [48], so that the influx of wealth and resources into historically marginalized communities does not come with more traffic pollution or with displacement of low-income or long-term residents.

## Supplementary Information

Below is the link to the electronic supplementary material.

ESM 1 (DOCX 341 KB)
