# Supplementary material for: Redlining, Community Wealth, and Air Pollution: A Tale of Three Cities—Boston, Nashville, and Detroit
Source: J Urban Health. 2026 Mar 11;103(1):120–34. doi: 10.1007/s11524-025-01052-0 (PMC13136452; doi:10.1007/s11524-025-01052-0)
Supplement: Supplementary file 1 — (DOCX 341 KB) [file 11524_2025_1052_MOESM1_ESM.docx]

**Electronic Supplementary Material**

Supplementary Table 1a: Average Annual PM_2.5_ (μg/m) between 2000-2016 (means and standard errors)

| **Redlining score category** | **2000** | **2001** | **2002** | **2003** | **2004** | **2005** | **2006** | **2007** | **2008** | **2009** | **2010** | **2011** | **2012** | **2013** | **2014** | **2015** | **2016** |
| --- | --- | --- | --- | --- | --- | --- | --- | --- | --- | --- | --- | --- | --- | --- | --- | --- | --- |
| **Boston** |  |  |  |  |  |  |  |  |  |  |  |  |  |  |  |  |  |
| D | 11.51 | 13.17 | 11.92 | 11.55 | 11.00 | 11.69 | 10.35 | 10.93 | 10.19 | 9.65 | 8.88 | 9.17 | 8.45 | 7.93 | 7.15 | 7.17 | 6.47 |
|  | (0.11) | (0.10) | (0.08) | (0.09) | (0.11) | (0.06) | (0.06) | (0.05) | (0.06) | (0.06) | (0.06) | (0.05) | (0.07) | (0.06) | (0.06) | (0.07) | (0.06) |
| C | 11.40 | 13.04 | 11.83 | 11.74 | 11.25 | 11.83 | 10.37 | 10.94 | 10.15 | 9.63 | 8.99 | 9.32 | 8.52 | 7.93 | 7.06 | 7.09 | 6.39 |
|  | (0.09) | (0.09) | (0.08) | (0.10) | (0.10) | (0.06) | (0.06) | (0.04) | (0.06) | (0.06) | (0.05) | (0.06) | (0.07) | (0.04) | (0.05) | (0.07) | (0.05) |
| B | 11.33 | 13.00 | 11.81 | 11.63 | 11.23 | 11.79 | 10.30 | 10.81 | 10.00 | 9.50 | 8.99 | 9.28 | 8.40 | 7.94 | 7.01 | 6.81 | 6.33 |
|  | (0.12) | (0.12) | (0.11) | (0.14) | (0.14) | (0.09) | (0.08) | (0.06) | (0.08) | (0.09) | (0.06) | (0.07) | (0.09) | (0.06) | (0.06) | (0.09) | (0.07) |
| Ungraded | 10.57 | 12.46 | 10.98 | 10.76 | 10.20 | 11.13 | 9.79 | 10.42 | 9.40 | 8.74 | 8.58 | 8.83 | 7.91 | 7.73 | 6.76 | 6.23 | 6.12 |
|  | (0.22 | (0.25) | (0.18) | (0.27) | (0.29) | (0.22) | (0.19) | (0.15) | (0.19) | (0.19) | (0.18) | (0.15) | (0.23) | (0.14) | (0.16) | (0.20) | (0.23) |
| A | 10.99 | 12.87 | 11.62 | 11.38 | 11.05 | 11.67 | 10.35 | 10.78 | 9.80 | 9.37 | 8.90 | 9.37 | 8.37 | 7.93 | 6.86 | 6.63 | 6.50 |
|  | (0.14) | (0.13) | (0.12) | (0.14) | (0.16) | (0.10) | (0.10) | (0.08) | (0.09) | (0.10) | (0.08) | (0.08) | (0.09) | (0.07) | (0.06) | (0.09) | (0.07) |
| Overall mean | 11.30 | 13.01 | 11.78 | 11.57 | 11.11 | 11.73 | 10.33 | 10.86 | 10.03 | 9.53 | 8.93 | 9.27 | 8.43 | 7.92 | 7.02 | 6.93 | 6.41 |
| Overall standard error | (0.05) | (0.05) | (0.05) | (0.06) | (0.06) | (0.04) | (0.04) | (0.03) | (0.04) | (0.04) | (0.03) | (0.03) | (0.04) | (0.03) | (0.03) | (0.04) | (0.03) |
| **Nashville** |  |  |  |  |  |  |  |  |  |  |  |  |  |  |  |  |  |
| D (Redlined) | 15.57 | 14.35 | 13.34 | 13.63 | 12.59 | 14.05 | 14.05 | 12.81 | 11.74 | 10.99 | 11.45 | 11.21 | 10.08 | 9.56 | 10.12 | 9.21 | 8.26 |
|  | (0.09) | (0.05) | (0.05) | (0.07) | (0.08) | (0.05) | (0.05) | (0.04) | (0.06) | (0.04) | (0.03) | (0.05) | (0.04) | (0.08) | (0.04) | (0.07) | (0.05) |
| C | 15.67 | 14.39 | 13.37 | 13.56 | 12.57 | 13.98 | 14.08 | 12.8 | 11.64 | 10.87 | 11.26 | 11.19 | 9.92 | 9.82 | 10.13 | 9.18 | 8.10 |
|  | (0.08) | (0.07) | (0.03) | (0.10) | (0.10) | (0.06) | (0.06) | (0.09) | (0.08) | (0.08) | (0.06) | (0.06) | (0.06) | (0.10) | (0.06) | (0.08) | (0.11) |
| B | 15.88 | 14.44 | 13.42 | 13.44 | 12.48 | 14.04 | 13.99 | 12.87 | 11.5 | 10.77 | 11.2 | 11.17 | 9.97 | 9.29 | 10.08 | 8.9 | 8.15 |
|  | (0.10) | (0.11) | (0.08) | (0.15) | (0.10) | (0.06) | (0.10) | (0.12) | (0.12) | (0.06) | (0.08) | (0.11) | (0.05) | (0.13) | (0.08) | (0.13) | (0.15) |
| A | 15.69 | 14.27 | 13.45 | 13.32 | 12.37 | 13.97 | 13.65 | 12.73 | 11.4 | 10.72 | 11.19 | 10.99 | 9.88 | 9.00 | 9.90 | 8.95 | 8.05 |
|  | (0.09) | (0.09) | (0.06) | (0.10) | (0.07) | (0.06) | (0.09) | (0.11) | (0.10) | (0.04) | (0.06) | (0.08) | (0.04) | (0.09) | (0.07) | (0.08) | (0.11) |
| Overall mean | 15.67 | 14.35 | 13.39 | 13.49 | 12.5 | 14.01 | 13.92 | 12.79 | 11.58 | 10.85 | 11.3 | 11.13 | 9.97 | 9.39 | 10.05 | 9.08 | 8.15 |
| Overall standard error | 0.05) | 0.04) | 0.03) | 0.05) | 0.04) | 0.03) | 0.04) | 0.04) | 0.05) | 0.03) | 0.03) | 0.04) | 0.02) | 0.06) | 0.03) | 0.05) | 0.05) |
| **Detroit** |  |  |  |  |  |  |  |  |  |  |  |  |  |  |  |  |  |
| D (Redlined) | 15.47 | 15.87 | 15.56 | 15.38 | 13.70 | 15.52 | 12.99 | 12.80 | 11.69 | 10.73 | 10.37 | 9.74 | 9.22 | 9.14 | 10.10 | 9.76 | 8.43 |
|  | (0.06) | (0.05) | (0.06) | (0.05) | (0.05) | (0.03) | (0.04) | (0.03) | (0.03) | (0.03) | (0.04) | (0.05) | (0.03) | (0.04) | (0.03) | (0.03) | (0.06) |
| C | 15.09 | 15.66 | 15.23 | 15.05 | 13.36 | 15.39 | 12.76 | 12.63 | 11.48 | 10.58 | 10.14 | 9.62 | 9.27 | 8.97 | 9.99 | 9.71 | 8.34 |
|  | (0.07) | (0.06) | (0.05) | (0.05) | (0.05) | (0.03) | (0.04) | (0.03) | (0.04) | (0.03) | (0.03) | (0.05) | (0.04) | (0.03) | (0.04) | (0.04) | (0.07) |
| B | 14.77 | 15.44 | 14.99 | 14.85 | 13.18 | 15.26 | 12.56 | 12.47 | 11.33 | 10.41 | 10.10 | 9.45 | 9.23 | 8.86 | 9.88 | 9.72 | 8.28 |
|  | (0.08) | (0.07) | (0.08) | (0.07) | (0.06) | (0.04) | (0.06) | (0.04) | (0.06) | (0.04) | (0.04) | (0.06) | (0.05) | (0.05) | (0.05) | (0.05) | (0.1) |
| Ungraded | 14.86 | 15.74 | 14.85 | 15.08 | 13.32 | 15.42 | 12.70 | 12.61 | 11.41 | 10.54 | 10.14 | 9.80 | 9.51 | 8.98 | 9.99 | 9.45 | 8.18 |
|  | (0.04) | (0.05) | (0.04) | (0.04) | (0.03) | (0.02) | (0.02) | (0.01) | (0.02) | (0.02) | (0.03) | (0.03) | (0.03) | (0.02) | (0.02) | (0.02) | (0.06) |
| A | 14.73 | 15.62 | 14.87 | 14.94 | 13.27 | 15.34 | 12.62 | 12.53 | 11.33 | 10.46 | 10.12 | 9.56 | 9.38 | 8.91 | 9.9 | 9.56 | 8.2 |
|  | (0.05) | (0.06) | (0.04) | (0.05) | (0.04) | (0.03) | (0.04) | (0.03) | (0.04) | (0.03) | (0.03) | (0.05) | (0.04) | (0.03) | (0.04) | (0.03) | (0.08) |
| Overall mean | 15 | 15.68 | 15.1 | 15.08 | 13.38 | 15.4 | 12.73 | 12.62 | 11.46 | 10.55 | 10.18 | 9.66 | 9.33 | 8.98 | 9.98 | 9.63 | 8.28 |
| Overall standard error | (0.04 | (0.05) | (0.04) | (0.04) | (0.03) | (0.02) | (0.02) | (0.01) | (0.02) | (0.02) | (0.03) | (0.03) | (0.03) | (0.02) | (0.02) | (0.02) | (0.06) |

Supplementary Table 1b: Average Annual NO_2_ (ppb) between 2000-2016 (means and standard errors)

| **Redlining score category** | **2000** | **2001** | **2002** | **2003** | **2004** | **2005** | **2006** | **2007** | **2008** | **2009** | **2010** | **2011** | **2012** | **2013** | **2014** | **2015** | **2016** |
| --- | --- | --- | --- | --- | --- | --- | --- | --- | --- | --- | --- | --- | --- | --- | --- | --- | --- |
| **Boston** |  |  |  |  |  |  |  |  |  |  |  |  |  |  |  |  |  |
| D | 39.07 | 39.20 | 35.54 | 35.69 | 31.39 | 33.27 | 31.00 | 31.41 | 33.18 | 29.24 | 28.79 | 32.93 | 25.45 | 26.67 | 26.51 | 26.66 | 22.61 |
|  | (0.32) | (0.38) | (0.32) | (0.39) | (0.62) | (0.25) | (0.27) | (0.23) | (0.28) | (0.24) | (0.29) | (0.33) | (0.22) | (0.36) | (0.28) | (0.31) | (0.28) |
| C | 38.17 | 38.41 | 34.58 | 33.79 | 31.00 | 32.33 | 30.44 | 30.34 | 32.36 | 28.28 | 27.79 | 31.65 | 24.80 | 25.10 | 25.00 | 24.55 | 21.47 |
|  | (0.30) | (0.35) | (0.28) | (0.26) | (0.43) | (0.24) | (0.28) | (0.22) | (0.29) | (0.21) | (0.26) | (0.33) | (0.22) | (0.30) | (0.24) | (0.33) | (0.22) |
| B | 36.48 | 36.29 | 33.71 | 33.28 | 30.77 | 31.52 | 29.36 | 29.40 | 31.07 | 27.45 | 26.53 | 30.02 | 23.94 | 24.30 | 24.48 | 23.96 | 20.80 |
|  | (0.38 | (0.47 | (0.35 | (0.42 | (0.60) | (0.36) | (0.44) | (0.36) | (0.43) | (0.34) | (0.39) | (0.49) | (0.34) | (0.49) | (0.43) | (0.45) | (0.34) |
| Ungraded | 32.45 | 31.99 | 30.45 | 29.51 | 26.14 | 27.70 | 25.58 | 25.49 | 26.37 | 23.41 | 22.55 | 25.35 | 20.75 | 20.25 | 20.79 | 20.77 | 18.11 |
|  | (0.76) | (1.18) | (1.06) | (1.14) | (1.21) | (0.84) | (0.92) | (0.92) | (1.06) | (0.88) | (0.90) | (1.16) | (0.87) | (1.07) | (1.11) | (1.11) | (0.80) |
| A | 35.21 | 35.19 | 32.72 | 33.48 | 30.51 | 31.14 | 28.17 | 28.61 | 29.88 | 26.60 | 25.18 | 29.10 | 23.28 | 23.40 | 23.78 | 23.74 | 20.63 |
|  | (0.40) | 0.56) | (0.53) | (0.62) | (0.82) | (0.49) | (0.49) | (0.49) | (0.53) | (0.46) | (0.43) | (0.55) | (0.37) | (0.50) | (0.50) | (0.53) | (0.38) |
| Overall mean | 37.25 | 37.30 | 34.11 | 33.91 | 30.77 | 31.98 | 29.73 | 29.88 | 31.59 | 27.83 | 27.07 | 30.89 | 24.33 | 24.79 | 24.85 | 24.62 | 21.31 |
| Overall standard error | (0.19) | (0.23) | (0.19) | (0.21) | (0.29) | (0.17) | (0.19) | (0.17) | (0.2) | (0.16) | (0.18) | (0.22) | (0.15) | (0.21) | (0.18) | (0.20) | (0.15) |
| **Nashville** |  |  |  |  |  |  |  |  |  |  |  |  |  |  |  |  |  |
| D | 36.32 | 34.09 |  |  | 28.7 | 32.03 | 33.02 | 33.92 | 28.46 | 25.36 | 24.93 | 26.02 | 24.9 | 19.42 | 24.17 | 23.91 | 22.58 |
|  | (0.38 | (0.20 |  |  | (0.25) | (0.25) | (0.38) | (0.15) | (0.09) | (0.18 | (0.45) | (0.21 | (0.28) | (0.25) | (0.19 | (0.47) | (0.52) |
| C | 34.89 | 33.77 |  |  | 27.84 | 31.78 | 32.41 | 34.18 | 28.68 | 25.13 | 24.9 | 25.5 | 23.94 | 18.92 | 23.44 | 22.65 | 21.33 |
|  | (0.54 | (0.16 |  |  | (0.43) | (0.39) | (0.60) | (0.24) | (0.14) | (0.29) | (0.35) | (0.26) | (0.36) | (0.39) | (0.26) | (0.69) | (0.63) |
| B | 34.66 | 33.29 |  |  | 28.5 | 32.04 | 32.36 | 33.18 | 27.71 | 24.36 | 25.01 | 24.56 | 23.76 | 19.23 | 23.09 | 22.32 | 20.57 |
|  | (0.31 | (0.18 |  |  | (0.44) | (0.33) | (0.60) | (0.16) | (0.18) | (0.28) | (0.38) | (0.35) | (0.47) | (0.36) | (0.47) | (0.80) | (0.93) |
| A | 33.51 | 31.98 |  |  | 26.77 | 30.11 | 29.19 | 30.44 | 24.91 | 21.74 | 22.91 | 22.89 | 22.16 | 17.59 | 20.01 | 19.94 | 17.07 |
|  | (0.46 | 0.49 |  |  | (0.64 | (0.52) | (0.83) | (0.74) | (0.74) | (0.64) | (0.55) | (0.56) | (0.43) | (0.48) | (0.78) | (0.76) | (1.05) |
| Overall mean | 34.91 | 33.25 |  |  | 27.9 | 31.38 | 31.6 | 32.77 | 27.27 | 24.02 | 24.3 | 24.71 | 23.68 | 18.72 | 22.56 | 22.18 | 20.3 |
| Overall standard error | (0.26 | (0.20) |  |  | (0.26) | (0.23) | (0.37) | (0.31) | (0.30) | (0.29) | (0.27) | (0.25) | (0.23) | (0.21) | (0.34) | (0.38) | (0.49) |
| **Detroit** |  |  |  |  |  |  |  |  |  |  |  |  |  |  |  |  |  |
| D | 41.29 | 39.38 | 36 | 37.35 | 30.71 | 34.56 | 29.16 | 28.4 | 27.09 | 27.13 | 25.15 | 29 | 26.4 | 24.24 | 25.93 | 26.63 | 24.39 |
|  | (0.19) | (0.13) | (0.14) | (0.14) | (0.17) | (0.09) | (0.07) | (0.02) | (0.06) | (0.08) | (0.05) | (0.14) | (0.09) | (0.12) | 0.05 | 0.08 | 0.09 |
| C | 40.04 | 38.6 | 35.45 | 36.38 | 29.54 | 33.89 | 28.76 | 28.32 | 26.71 | 26.64 | 24.97 | 28.12 | 26.38 | 24.29 | 25.78 | 26.54 | 23.99 |
|  | (0.18) | (0.15) | (0.13) | (0.13) | (0.13) | (0.08) | (0.07) | (0.03) | (0.05) | (0.07) | (0.04) | (0.13) | (0.09) | (0.14) | 0.05 | 0.09 | 0.1 |
| B | 39.54 | 38.54 | 34.73 | 35.99 | 29.04 | 33.49 | 28.48 | 28.34 | 26.45 | 26.51 | 24.75 | 27.71 | 26.25 | 24.1 | 25.72 | 26.44 | 23.94 |
|  | (0.24) | (0.20) | (0.17) | (0.16) | (0.17) | (0.13) | (0.09) | (0.04) | (0.07) | (0.07) | (0.07) | (0.15) | (0.13) | (0.19) | 0.06 | 0.12 | 0.12 |
| Ungraded | 38.32 | 37.8 | 34.25 | 36.07 | 29.24 | 33.39 | 28.1 | 28.02 | 25.87 | 25.77 | 24.37 | 26.58 | 26.15 | 24.28 | 25.61 | 25.85 | 23.57 |
|  | (0.12) | (0.10) | (0.09) | (0.08) | (0.10) | (0.06) | (0.09) | (0.06) | (0.06) | (0.06) | (0.07) | (0.09) | (0.07) | (0.12) | 0.04 | 0.08 | 0.08 |
| A | 38.86 | 38.27 | 34.49 | 35.92 | 29.13 | 33.55 | 28.33 | 28.25 | 26.16 | 26.13 | 24.57 | 26.91 | 26.2 | 24.2 | 25.54 | 26.17 | 23.57 |
|  | (0.16) | (0.16) | (0.10) | (0.11) | (0.12) | (0.08) | (0.08) | (0.05) | (0.06) | (0.06) | (0.07) | (0.12) | (0.09) | (0.15) | 0.05 | 0.1 | 0.1 |
| Overall mean | 39.58 | 38.49 | 34.99 | 36.36 | 29.56 | 33.78 | 28.56 | 28.25 | 26.44 | 26.41 | 24.75 | 27.64 | 26.28 | 24.23 | 25.72 | 26.3 | 23.88 |
| Overall standard error | (0.09) | (0.07) | (0.06) | (0.06) | (0.07) | (0.04) | (0.04) | (0.02) | (0.03) | (0.03) | (0.03) | (0.06) | (0.04) | (0.06) | 0.02 | 0.04 | 0.04 |

Supplementary Figure 1: Distribution of Social Vulnerability Index (SVI) by redlining score category and city.

SVI data in 2000 represent social vulnerability ascertained for that single year, whereas the SVI data that included the year 2016 (published in 2018) represented a 5-year window covering/summarizing data from 2012 through 2016.


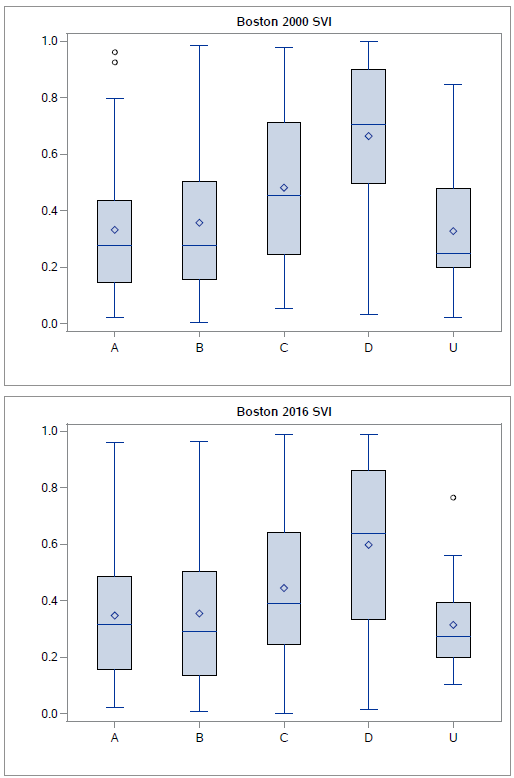

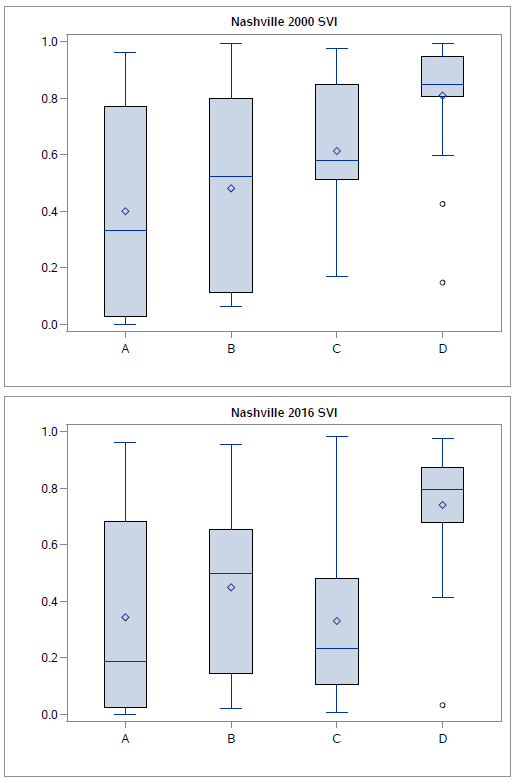

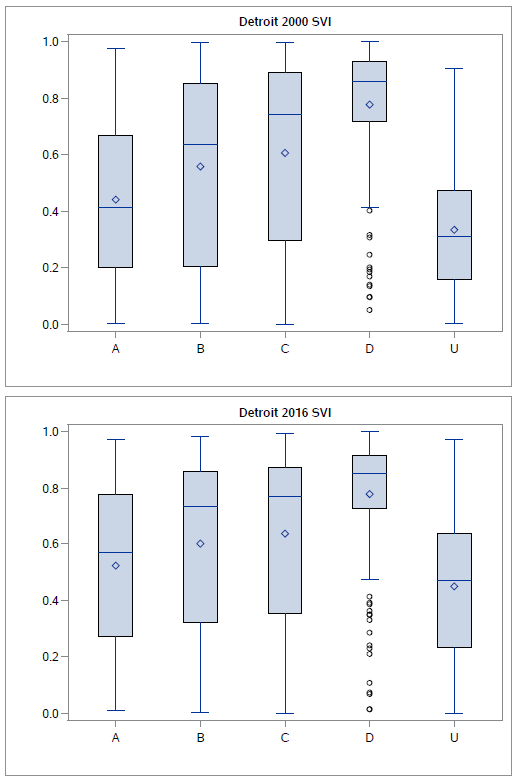


**Supplementary Methods:**

1. **Nashville NO_2_ Rationale for exclusion of values from 2002-2003**


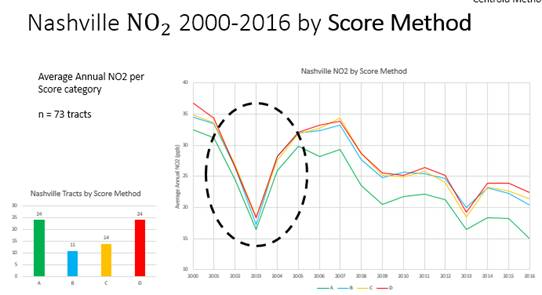


The yearly change in NO2 in Nashville for 15 of the 17 years varied between approximately -2% to 5%.

For 2002 the drop was about 23% (from ~34 to ~26), and for 2003 it was down another 38% (~26 - ~16).  Examining monthly trends between normal years 2001 and 2005 against 2003, 2003 shows an extreme drop to values near zero for much of June, July, and all of August. The rest of the months do not seem as adversely affected; however they exhibit extremes such as months with abnormally low fluctuation in the readings (late October to mid-November), and months with high fluctuation (April). We consider the 2002-03 data outliers, with no apparent reason in terms of known actual changes in pollutions sources for this drastic drop, and with PM pollution in this same period not dropping precipitously. Local historical data related to sensor measurement or data processing was not available at the time of the writing of this manuscript.

1. **Accounting for changes in census tract boundaries between 2000 and 2010**

To determine the 2000 tracts that correspond to the 2010 tracts identified for each city we used a crosswalk. This crosswalk was created by first calculating population centroids for each 2010 tract polygon. These 2010 tract centroids were overlaid with the 2000 tract polygons using a spatial join process in ArcGIS Pro. The result was a new field containing the 2000 tract that each of the 2010 tract centroid fell into. This list of corresponding 2000 tracts was used to extract the SES and SVI data from 2000.
